# Comparison of the efficacy of second and third generation lentiviral vector transduced CAR CD19 T cells for use in the treatment of acute lymphoblastic leukemia both in vitro and in vivo models

**DOI:** 10.1371/journal.pone.0281735

**Published:** 2023-02-13

**Authors:** Piamsiri Sawaisorn, Korakot Atjanasuppat, Kitipong Uaesoontrachoon, Parin Rattananon, Worapapar Treesuppharat, Suradej Hongeng, Usanarat Anurathapan

**Affiliations:** 1 Division of Hematology and Oncology, Department of Pediatrics, Faculty of Medicine Ramathibodi Hospital, Mahidol University, Bangkok, Thailand; 2 Genepeutic Bio, Co. Ltd., Bangkok, Thailand; 3 Thammasat University Research Unit in Mechanisms of Drug Action and Molecular Imaging, Drug Discovery and Development Center, Office of Advanced Science and Technology, Thammasat University, Pathum Thani, Thailand; The University of Burdwan, INDIA

## Abstract

T cells genetically engineered to express a chimeric antigen receptor (CAR) specifically binding to a CD19 antigen has become the frontline of hematological malignancies immunotherapy. Their remarkable antitumor effect has exerted complete remission in treating B-cell malignancies. Although successful patient treatment has been shown, improvement to the structure of CAR to enhance its safety and efficacy profile is warranted. Transduction with a lentiviral vector (LVV) leading to the expression of CARs is also a critical step in redirecting T cells to target specific tumor antigens. To improve the efficacy of CD19 CARs in this study, the transduction ability of second and third generations LVV were compared. Ex vivo expansion of CD19 CARs T cells from healthy donors’ peripheral blood mononuclear cells was performed after transduction of T cells with second and third generations LVV. Transduction efficacy of transduced T cells was determined to show a higher percentage in the third generations LVV transduced cells, with no changes in viability and identity of cells characterized by immunophenotyping. Testing the cytotoxic capacity of third generations LVV-transduced T cells against target cells showed higher reactivity against control cells. Cytokine expression was detected on the CD19 CARs T cells, suggesting that these cells limit in vitro growth of B-cell leukemia via secretion of the pro-inflammatory cytokine IFN γ. To investigate whether the third generation LVV transduced T cells can limit CD19 lymphoma growth in vivo, an analysis of tumor burden in a mouse model assessed by bioluminescence imaging was performed. We found that, in the presence of CD19 CARs T cells, the level of tumor burden was markedly reduced. In addition, an increase in the length of survival in mice receiving CAR-CD19 T cells was also observed. This suggests that transduction with third generations LVV generate a functional CAR-CD19 T cells, which may provide a safer and effective therapy for B-cell malignancies.

## Introduction

Acute Lymphoblastic Leukemia (ALL) is the most common type of leukemia in children worldwide [1]. Most ALL arises in healthy individuals and is characterized by chromosomal abnormalities and genetic alterations involved in the differentiation and proliferation of lymphoid precursor cells. The development of intensified chemotherapy protocols substantially improved the outcome of patients with ALL, particularly in children (1–14 years) and also in adolescents and young adults (15–39 years) [2]. In the past, ALL was intractable, but now the survival probability is as high as 80–90%. The treatment is modified based on biological characteristics (e.g., aneuploidy and translocation) and initial treatment response, which is assessed by the minimal residual disease (MRD) [3].

Treatment for pediatric ALL typically consists of induction therapy with steroids, vincristine, and asparaginase with or without anthracycline, followed by multi-agent consolidation, including high-dose methotrexate and re-induction therapy. After consolidation, less intensive maintenance therapy is required for 1–2 years to maintain an event-free survival [4]. Despite responding well to chemotherapy regimens, about 20% of children will relapse following treatment [5–8]. With significant attempts to optimize treatment, the overall survival of children with relapsed ALL remains less than 50% [9–11]. More recently, new immunotherapeutic strategies, such as monoclonal antibodies, CAR T cells and treatments targeting patient-specific genetic changes are being developed and are expected to change the options for ALL treatments [2].

CAR T cell therapy represents a significant advancement in personalized cancer treatment. In this strategy, a patient’s T cells or T cells from their hematopoietic stem cell (HSC) donor are genetically engineered via a lentiviral vector to express a synthetic receptor that binds to a tumor antigen. This binding is mediated by the expression of a single-chain protein consisting of an extracellular antigen-binding domain derived from a monoclonal antibody and the T-cell receptor’s signaling domain(s). CAR T cells are then expanded in the laboratory for clinical use and infused back into the patient, where they recognize and destroy target cells with enhance specificity, including chemotherapy resistant ALL cells. Therefore, the active and essential ingredient in CAR T cell therapy is the lentiviral vector used to modify T cells genetically. In general, lentiviruses require several significant genes, including Gag, Pol, Env, and the accessory genes, for their function and survival [12]. The lentiviral vector (LVV) used for gene therapy is developed and divided into different generations depending on the separated packaging plasmid that potentially confers a better safety profile and the promoter used. Currently, with the lack of accessory genes, the second and third generations of LVV are the most used. The main differences between the second and third generations of LVV are that the packaging plasmid for the latter is divided into two plasmids, one encoding Gag and Pol and the other encoding Rev, and the promoter used has 5’ LTR (long terminal repeat) deleted. Moreover, the deletion of the Tat gene, accessory virulence factors required for viral replication, and the creation of self-inactivation (SIN) by disrupting the promoter region of the LTR lead to improvement in the safety profile of the third generation LVV [13]. With the effectiveness and better safety profile of the different generations of LVV, several clinical trials have used LVV-transduced cells for the potential treatment of various genetic diseases [14–16]. The previous study has shown that using second-generation anti-CD19 scFv-CD28-CD3ζ CAR-T cells transduced with the second-generation LVV conferred significant efficacy *in vitro* and patients under compassionate grounds [17]. In the present study, we sought to compare the effects of transduced T cells by the second and third generations LVV *in vitro* and *in vivo*. The study demonstrated that the third-generation LVV produced a superior efficacy as determined by using lower MOI in gaining similar effects in all analytical parameters tested compared to second-generation LVV (MOI of 5 vs. 20). In addition, the use of third-generation LVV also showed a reduction in tumor burden and increased survival in the animal model of leukemia. These findings warrant using third-generation LVV in the CAR T production for a clinical trial for ALL treatments.

## Materials and methods

### Cell lines and PBMCs samples

The Raji cell line (Burkitt’s lymphoma) and Jurkat cell line (acute T-lymphoblastic leukemia) were kindly provided by Assistant Professor Dachit Nilubol and Professor Kovit Pattanapanyasat. Both cell lines were grown in RPMI-1640 medium (Hyclone, USA) with 10% FBS (Gibco, USA) and 1% penicillin/streptomycin (Gibco, USA).

Human peripheral mononuclear cells (PBMCs), obtained from healthy donors, were isolated by density gradient centrifugation using Ficoll-Paque (Corning, USA). The PBMCs were then cultured in RPMI 1640 medium with 10% fetal bovine serum and 1% penicillin/streptomycin.

For the generation of allogeneic cells, PBMCs were stimulated with 5 μg/ml phytohemagglutinin (PHA) (Invitrogen, USA.) in RPMI-1640 medium supplemented with 10% FBS, 1% penicillin/streptomycin and 100 U/ml recombinant IL-2 (PeproTech, USA). The allogeneic cells were cultured for seven days, during which the medium and IL-2 were replaced. The medium and IL-2 of viable cells were changed every 2–3 days. All cells were maintained at 37 °C with 5% CO_2_. The study was approved by the Ethical Clearance Committee on Human Rights Related to Research Involving Human Subjects, Faculty of Medicine Ramathibodi Hospital, Mahidol University (MURA2020/879). The written consents were obtained from all the participants involved in this study.

### T cells transduction

Isolated PBMCs were activated using anti-human CD3 (OKT3) and anti-human CD28 antibody (CD28.2) (eBioscience, USA) at the final concentration of 0.5-1x10^6^ cell/well in a 24-well culture plate. Recombinant IL-2 was added at a final concentration of 200 IU/ml and incubated for 72 hours. Activated T cells were transduced overnight at the final concentration of 0.5-1x10^6^ cell/well in a retronectin coated 24-well culture plate (20 ug/ml of retronectin in DPBS for 2 hours, room temperature) using either second (In house) or third generation LVV (VIVE Biotech, Spain). Transduced T cells were then expanded for seven days in the presence of 200 IU/ml of recombinant IL-2. Cells were then harvested and stored in the gas phase of the liquid nitrogen until used.

### DNA extraction and transduction efficiency

After transduction and cultivation, transduced and non-transduced CD19 CAR T cells were collected and extracted for genomic DNA using a GenUP^™^ gDNA Kit (Biotechrabbit, Germany). Genomic DNA was checked for purity using the NanoDrop Spectrophotometer (Thermo Fisher Scientific, USA). Real-time PCR was performed using a CFX96^™^ Real-Time PCR Detection System (Bio-Rad, USA) to determine the copy number of the transgene, CD3ζCD28, in the genomic DNA. Relative gene expression of the transgene was measured using SYBR^™^ Green Master Mix Kit (Bio-Rad, USA) based on a generated standard curve. All measurements were performed in duplicate and following the manufacturer’s instructions.

### Cytotoxicity assay

The cytotoxicity assay was performed using a flow cytometry-based analysis with three target cell lines (Raji, Jurkat, and allogeneic cells). All target cells were labeled with carboxyfluorescein diacetate succinimidyl ester (CFSE) (Invitrogen, USA) at 1 μM. Effector cells, transduced and non-transduced T cells, were co-cultured for 24 hours with all target cells at ratios of 20:1 (effector: target). Only target cells were used as a control to assess spontaneous death. After incubation, cells were collected and stained with 5 μl of 7-AAD (7-amino-actinomycin D) (eBioscience, USA). Analysis was performed using a Navios flow cytometer (Beckman Coulter, USA) with Navios software (Beckman Coulter, USA). The CFSE and 7-AAD positive cell population were used to indicate target cell death. The percentage of cytotoxic activity was calculated based on the following equation:

$$\text{Cytotoxicity}\;\left(\%\right)=\left[\left(\text{Target}\;\text{cell}\;\text{deaths}-\text{spontaneous}\;\text{deaths}\right)/\left(100-\text{spontaneous}\;\text{deaths}\right)\right]\times 100$$

### Immunophenotyping

After the transduction of T cells, cell surface staining to identify the identity of cells was performed. Briefly, cells were incubated at 4°C for 15 mins with a combination of antibodies as follows before flow cytometric (FACS) analysis: anti-CD3-APC (UCHT1), anti-CD4-PerCP (VT4), anti-CD45RA-PE (HI100), anti-CD45RO-PE (EH12.2H7) (all were purchased from BD Biosciences, USA), anti-CD8-APC-Vio^®^ 770 (REA734) (Miltenyi Biotec, USA), and anti-CD62L-PEcy7 (HP-MA4) (BioLegend, USA). The data acquisition was performed on a Navios flow cytometry instrument using the Navios software. The acquired data were analyzed with Kaluza software (Beckman Coulter, USA). Stained control samples were gated according to the fluorescence minus one technique.

### Measurement of Interferon-gamma (IFN- γ)

The level of pro-inflammatory cytokine IFN- γ was investigated. Supernatants were collected from co-cultivation between CAR-CD19 T cells with three different target cell lines after 24 hours and then proceeded for IFN- γ level detection via LEGENDplex^™^ using HU Essential Immune Response Panel (13-plex) (Biolegend, USA). The data acquisition was performed on a Navios flow cytometry instrument using the Navios software. The acquired data were analyzed using LEGENDplex software (Biolegend, USA) based on a standard curve. All measurements were performed in duplicate and by the manufacturer’s instructions.

### Detection of replication-competent lentivirus (RCL)

To assess RCL, the genomic DNA of non-transduced T cells was spiked with PMD2G plasmid in a 10-fold dilution to obtain a standard curve (10^0^ to 10^4^ copies). DNA was extracted using a GenUP^™^ gDNA Kit (Biotechrabbit, Germany) and checked for purity using the NanoDrop Spectrophotometer (Thermo Fisher Scientific, USA). Real-time PCR was performed on a CFX96^™^ Real-Time PCR Detection System (Bio-Rad, USA) using the Taqman probe technique to determine the copy number of the VSV-G gene. All measurements were performed in duplicate and per the manufacturer’s instructions.

### Animals

The animal experiments specified in this study were approved by the Thammasat University Animal Committee per the Thailand Council on Animal Care (Approval ID#005/2021). C.B.17 mice were purchased from Nomura Siam International. The animals were acclimatized upon arriving at the animal facility for seven days, housed in cages with up to 5 mice per cage. During the study, 12-hour light/12-hour dark cycles were maintained. All tests were performed during the light cycle phase. The room temperature was maintained between 22±1°C. Food and water were available ad libitum for the duration of the study. Mice were identified by ear tags. C.B.17 mice were sorted into treatment groups by body weight after acclimation. After the C.B.17 mouse weight was obtained, the average body weight of each cage was calculated. Randomization was then performed by using the average body weight of the cage. The mice were sorted into groups so that each group had a similar average body weight (<5% difference in average body weight between groups).

### Treatment

Mice received either vehicle or test article formulations via intravenous injection to the lateral tail vein. Twenty minutes (20 min) before the dosing time, a mouse was removed from the cage, anesthetized via isofluorane, and placed on the heating pad with its tail exposed. The needle (28-gauge insulin needle, Terumo, USA) was inserted into the lateral tail vein near the distal portion of the tail. The vehicle or test article was administered as a slow bolus. The pressure was applied over the injection site for a few moments. All animals were monitored for up to 30 minutes after the injection to ensure no adverse effects.

### Bioluminescence

To examine ALL-tumor growth in animals bearing RajiFFLuc, mice were monitored by In Vivo Imaging System MX FX PRO (Carestream Health Inc., Rochester, NY, USA) with Bruker Molecular Imaging software version 7.1.3.20550 (Bruker, Billerica, MA, USA). Two weeks after the tumor injection, a baseline scan was performed before treatment. Mice were infused by intraperitoneal injection with 150 mg/kg D-luciferin potassium salt (Xenolight^™^, Perkin Elmer, Boston, MA, USA) suspended in 200 μl DPBS. After 15 minutes, mice were imaged under 3% isoflurane anesthesia. Images were acquired on a 25-cm field of view at a medium binding level within 3 minutes of exposure time. Regular imaging started six weeks after the treatment injection. The bioluminescence intensity was analyzed and reported as the means of photon/sec/interior area (mean P/s/mm^2^).

### Statistical analysis

The results were expressed as mean ± SEM of 3 independent samples and analyzed using a two-tailed, paired t-test to compare the significance of differences between groups. One-way ANOVA with Bonferroni’s multiple comparison test was used to compare groups versus controls. A Log-rank test was used to test survival data. All tests were performed using GraphPad Prism version 8 (GraphPad Software). P<0.05 was considered statistically significant.

## Results

### Viability of transduced cells

**Fig 1:**
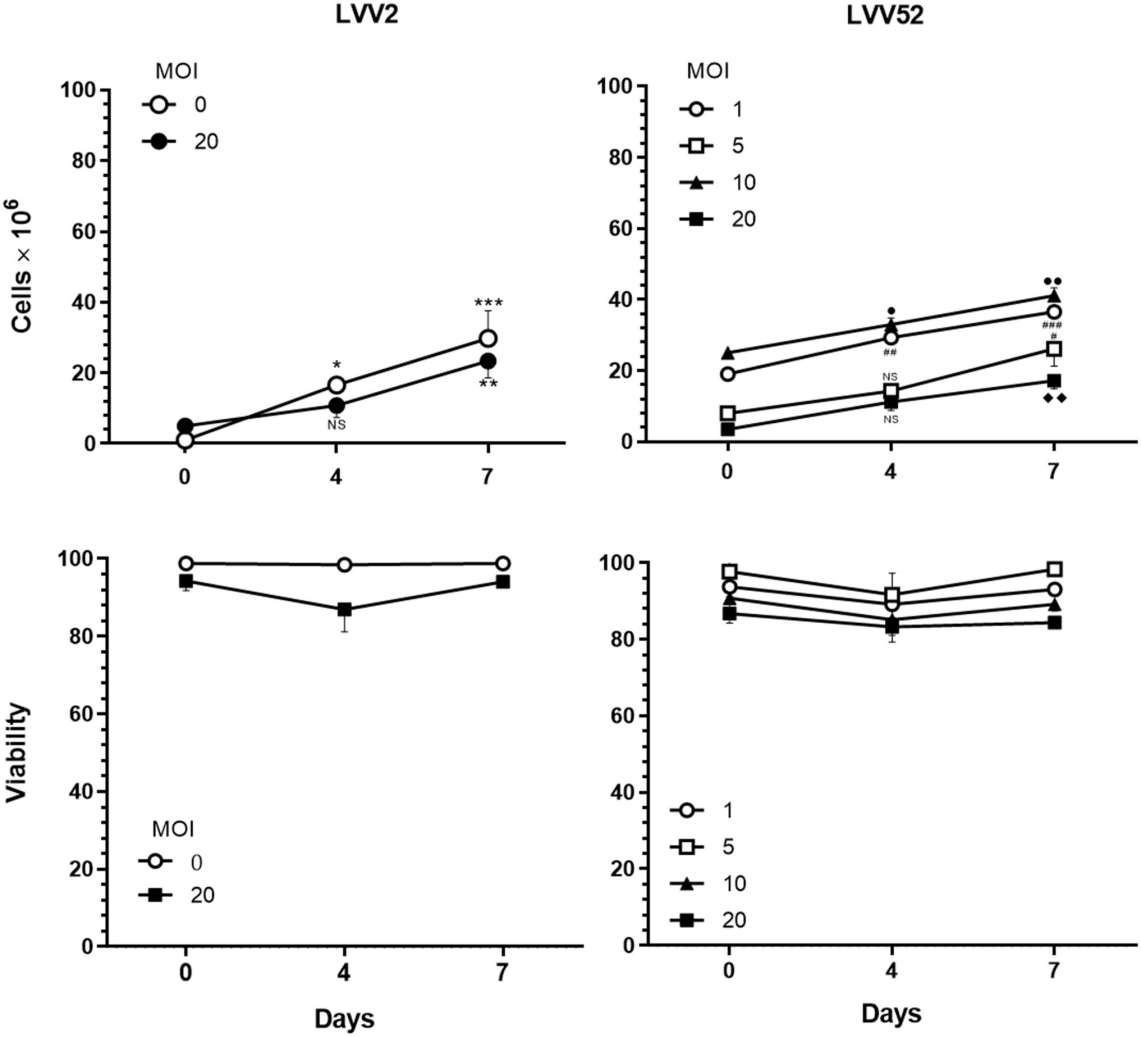
Cell expansion and viability of PBMC following transduction with LV52. PBMCs from 3 donors were transduced with the LVV2 (left panels) or LV52 (right panels) using the indicated MOIs. The upper panels showed the total cells recovered, and the lower panels showed the viability of the cultures at the indicated time points. The data shown are the mean ± SEM of 3 independent experiments. *p = 0.0122, **p = 0.0016, ***p = 0.0001, #*p* = 0.0177, ##*p* = 0.0052, ###*p* = 0.0003, ●*p* = 0.0477, ●●*p* = 0.0015, ◆◆*p* = 0.0072; one-way ANOVA.

Third-generation LVV (LV52) is as efficient in producing viable transduced T cells as second-generation LVV. At the end of cultivation, the transduced T cells were expanded similarly across all treatment groups, with more than 90.0% viable cells. The second generation LVV (LVV2) transduced T cells were expanded and reached the number of 2.135 × 10^7^ cells/mL at the MOI of 20 (range 1.735–2.650 × 10^7^ cells/mL; Fig 1A and 1C). On the other hand, T cells transduced with LV52 at MOI of 1 had 2.052 × 10^7^ cells/mL (range 1.830–2.355 × 10^7^ cells/mL), MOI of 5 had 2.113 × 10^7^ cells/mL (range 1.350–3.020 × 10^7^ cells/mL), MOI of 10 had 1.907 × 10^7^ cells/mL (range 1.545–2.300 × 10^7^ cells/mL), MOI of 20 had 1.668 × 10^7^ cells/mL (range 1.220–2.020 × 10^7^ cells/mL) and mock T cells had 2.980 × 10^7^ cells/mL (range 2.085–4.54 × 10^7^ cells/mL; Fig 1B and 1D). This finding suggests that the current LV52 did not affect the growth of the T cells and produced comparable viability results to cells transduced with LVV2.

### Immunophenotyping of CAR T cells

**Fig 2:**
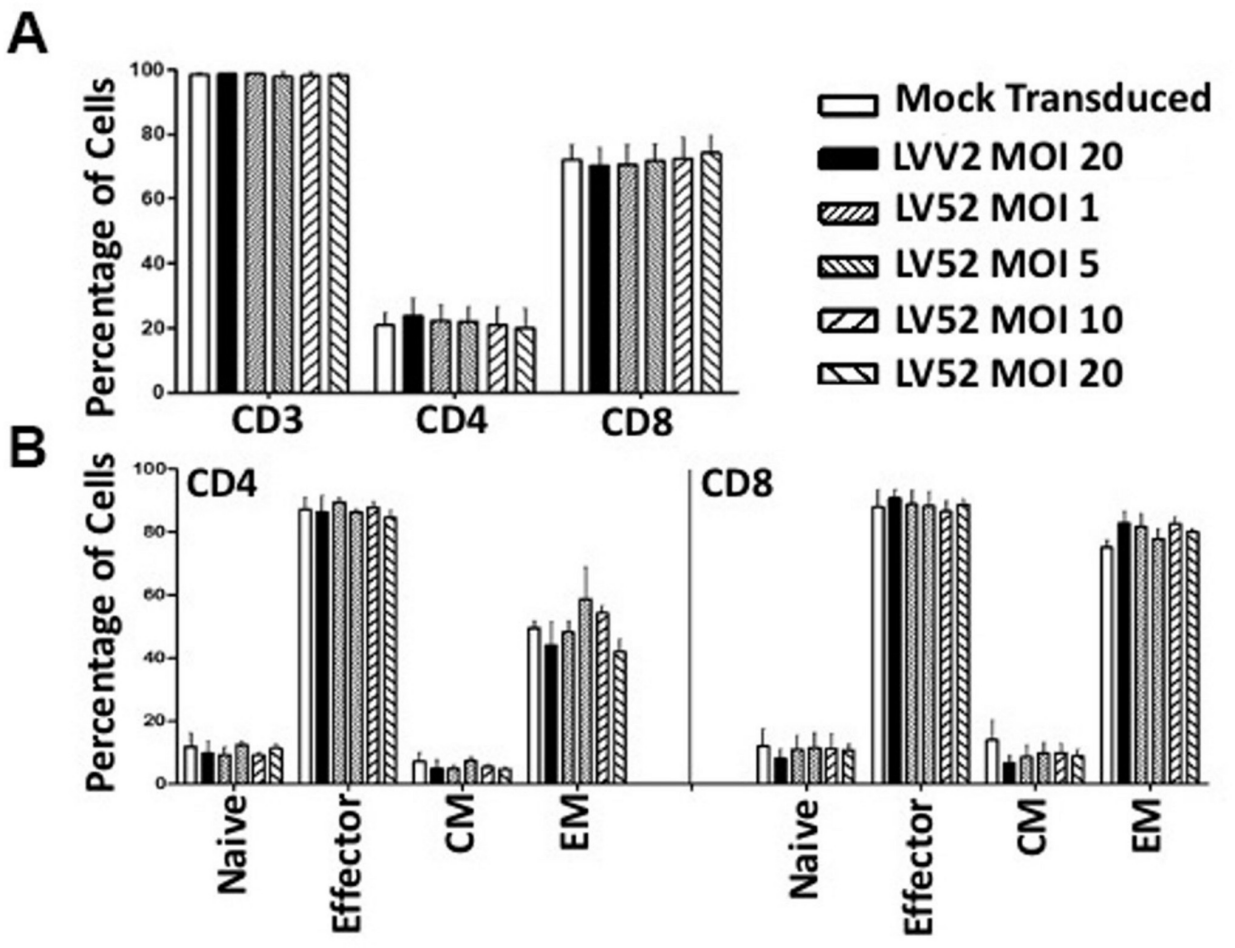
The phenotype of LV52 transduced cells. PBMCs from 3 donors were transduced as indicated and immuno-phenotyped by multicolor flow cytometry. (A) The proportion of T cells (CD3), helper T cells (CD4), and effector T cells (CD8). (B) The proportion of T cell subsets with effector/memory function within the CD4+ and CD8+ populations. The data shown are the mean ± SEM of 3 independent experiments.

CD19 CAR T cells were harvested, and their phenotype was determined using multicolor flow cytometry after seven days of culture. All the treatments tested showed that most cells were CD8+ regardless of the transduction vector or MOI used (Fig 2A). Further analysis revealed the presence of naïve (CD45RA+CD62L+), effector (CD45RA+CD62L-), central memory (CD45RO+CD62L+), and effector memory (CD45RO+CD62L-) cells. The CD8+ CD19 CAR T cells were predominately CD8+ effector memory cells (Fig 2B), with a similar distribution across treatment groups: 82.71% (range 75.46–88.81% for LVV2 transduced T cells, as compared to 81.59% (range 74.80–90.08%), 77.75% (range 72.79–84.01%), 82.46% (range 77.65–85.61%) and 80.03% (range 78.12–81.39%) for cells transduced with LV52 at MOIs of 1, 5, 10 and 20 respectively. Mock transduced cells were 75.04% (range 71.03–79.32%) CD8+ effector memory T cells (Fig 2B).

### CAR-CD19 expression level in transduced T cells

**Fig 3:**
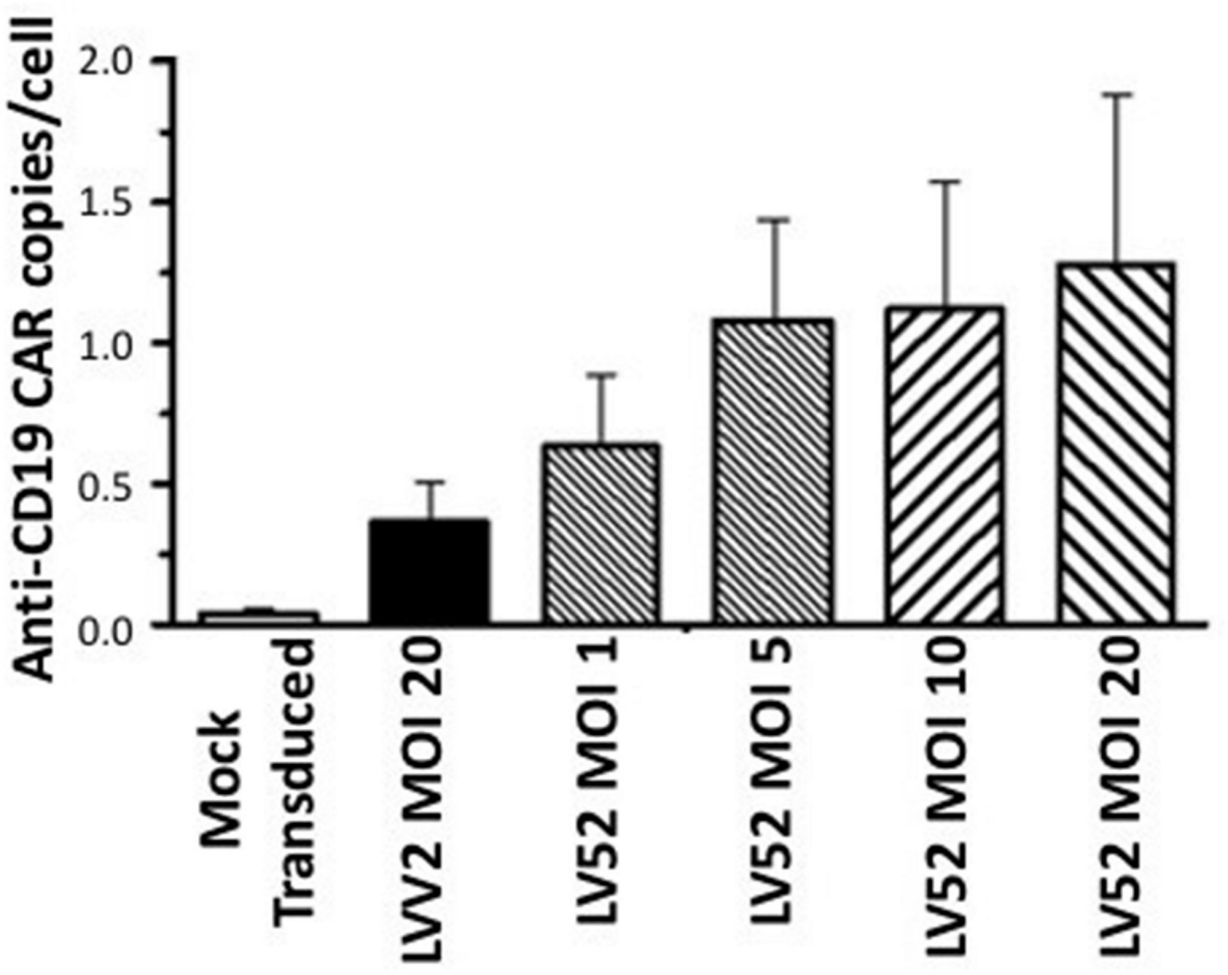
Efficacy of transduction of T cells with anti-CD19 CAR. PBMCs were mock transduced or transduced with LVV2 or LV52 lentiviral vectors using a range of MOI as indicated. Cells were assessed for anti-CD19 CAR expression using qPCR, and the number of transgene copies was expressed as the average number detected in each cell. Bars indicate the mean ± SEM of 3 independent experiments.

Anti-CD19 CAR expression was assessed by qPCR to determine the transgene copy number by detecting the following components: the CD28 transmembrane, the CD28 signaling domain, and a CD3ζ-derived signaling domain. The copy number per cell was calculated. The mean number of copies/cell was 0.04, 0.36, 0.63, 1.08, 1.12, and 1.30 in mock transduced T cells, T cells transduced with LVV2, and T cells transduced with LV52 at an MOI of 1, 5, 10 and 20, respectively (Fig 3). This data demonstrates the efficient transduction of T cells using the third-generation LVV.

### Cytotoxicity assay for CAR-CD19 T cells

**Fig 4:**
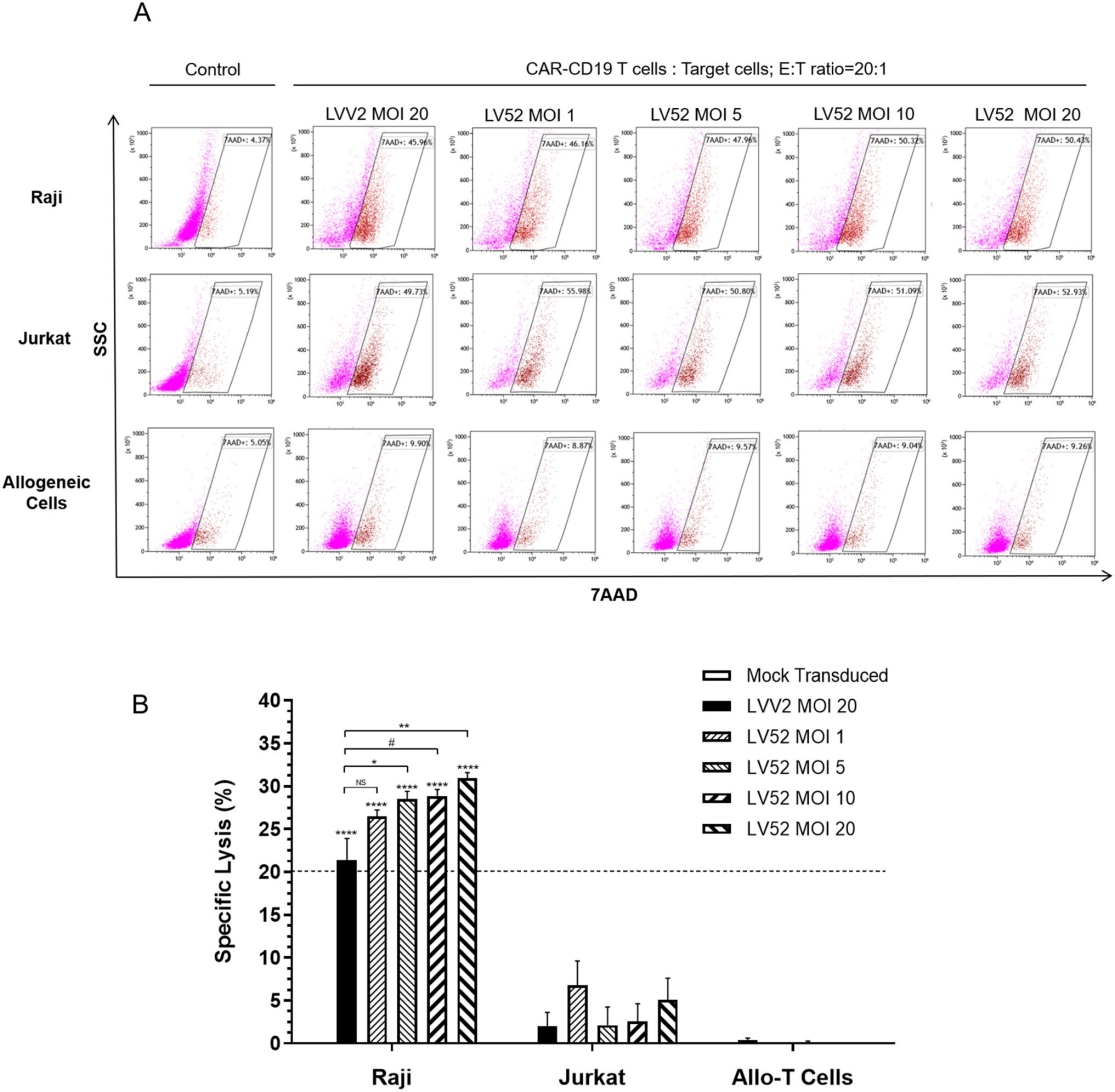
CAR-CD19 T cells specifically kill CD19-expressing cells. CAR-CD19 T cells were incubated with the indicated target cells at an E: T ratio of 20:1 for 24 hours, and the viability of target cells was assessed by flow cytometry using a CFSE-7AAD-based cytotoxic assay. (A) Representative flow cytometry panels with CFSE-7-AAD-based cytotoxic assay from one of three independent experiments. (B) The mean ± SEM of normalized cytotoxicity from 3 independent experiments is shown. *p = 0.0206, **p = 0.0018, #*p* = 0.0136; paired t-test. ****p<0.0001; one-way ANOVA.

The ability of transduced T cells to specifically kill CD19-expressing cells was demonstrated using cytotoxicity assays. These experiments were performed using three target cell types, Raji (CD19 expressing), Jurkat (CD19 negative; to determine the specificity), and allogeneic cells. The target cells were stained with Carboxyfluorescein Succinimidyl Ester (CFSE) and then co-cultured with the CAR-CD19 T cells or mock transduced T cells for 24 hours at an effector: target ratio of 20:1. Harvested cells were stained with 7-Aminoactinomycin D (7-AAD) and analyzed by flow cytometry. The LVV2 (positive control) and LV52 transduced CAR-CD19 T cells were used as the effector cells. All the CAR-CD19 T cells exhibited higher cytotoxicity against Raji cells than Jurkat cells or allogeneic PBMCs. The mean percentage of specific lysis after normalization to the mock transduced T cells for the LVV2 cells was 21.4%. The specific lysis of Raji cells by the LV52 transduced T cells was 26.5%, 28.5%, 28.9%, and 30.9% at MOIs of 1, 5, 10, and 20, respectively. Notably, there was no evidence of specific lysis of allogeneic cells (Fig 4).

### Level of interferon (IFN)-γ production by transduced T cells

**Fig 5:**
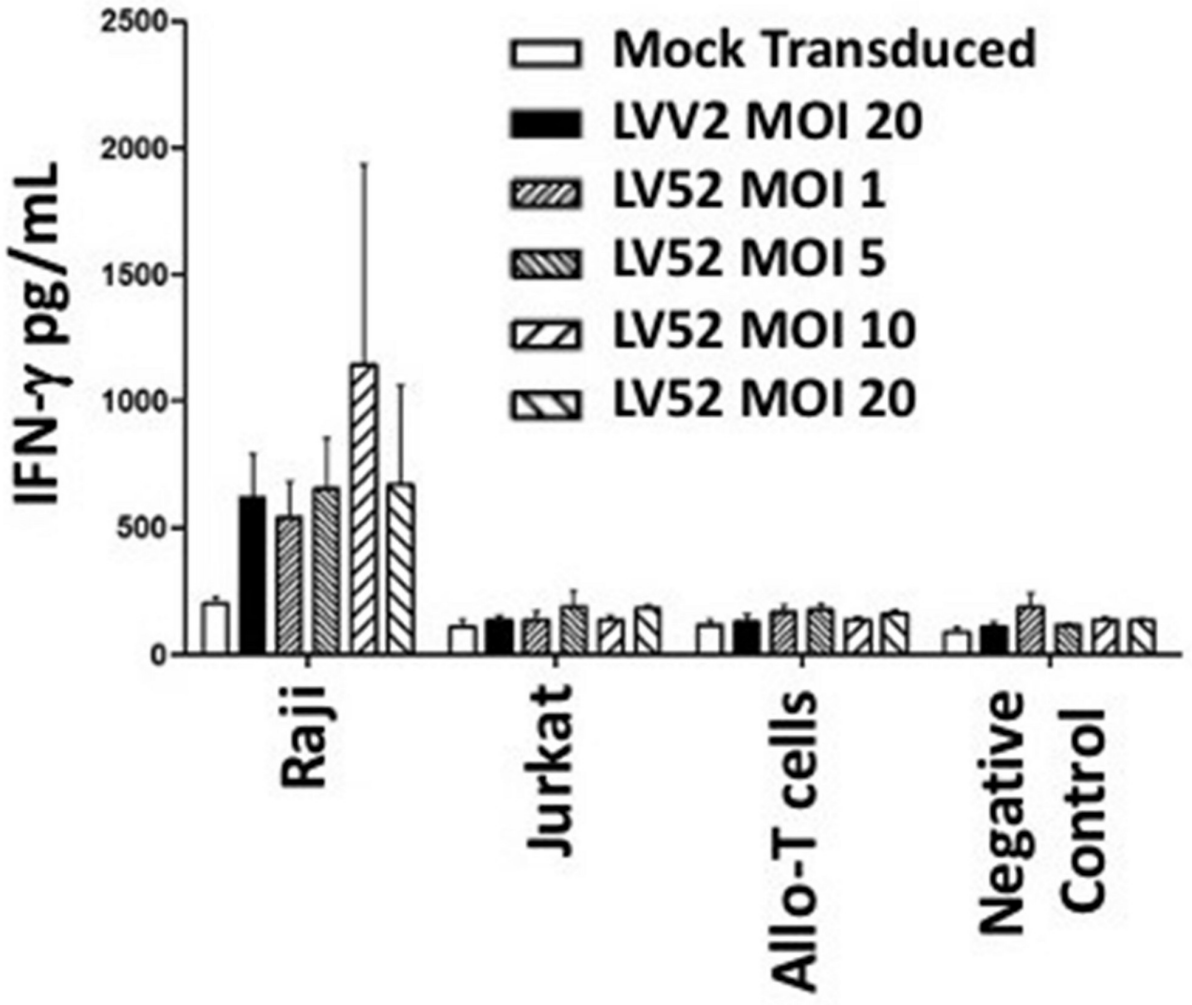
IFN-γ production by CAR-CD19 T cells. The concentration of IFN-γ in culture supernatants following 24 hours of co-culture between the indicated cell types and mock transduced or T cells transduced with LVV2 or LV52 at the indicated MOI using LEGENDplex^™^. The data shown are the mean ± SEM of 3 independent experiments.

CAR-CD19 T cells also release the pro-inflammatory cytokine, IFN-*γ*, which enhances cytotoxicity (Burke JD, Young, 2019). Therefore, the production of IFN-γ by LVV2 and LV52 transduced T cells when co-cultured with CD19-expressing cells was investigated. Supernatants were collected from co-cultures of CAR-CD19 T cells with three different target cell types after 24 hours incubation. The concentration of IFN-γ was determined using LEGENDplex^™^ via HU Essential Immune Response Panel (13-plex). IFN-γ production was increased in the CAR-CD19 T cells produced using both LVV2 and LV52 co-cultured with Raji cells. IFN-γ concentrations produced by mock transduced T cells were 200 pg/mL, while CAR-CD19 T cells made using the LVV2 (MOI of 20) produced 619 pg/mL of IFN-*γ*. CAR-CD19 T cells produced using LV52 at MOIs of 1, 5, 10, and 20 had 539, 653, 1,143, and 669 pg/mL, respectively (Fig 5).

### The ability of CAR-CD19 T cells to induce tumor suppression in mice

**Fig 6:**
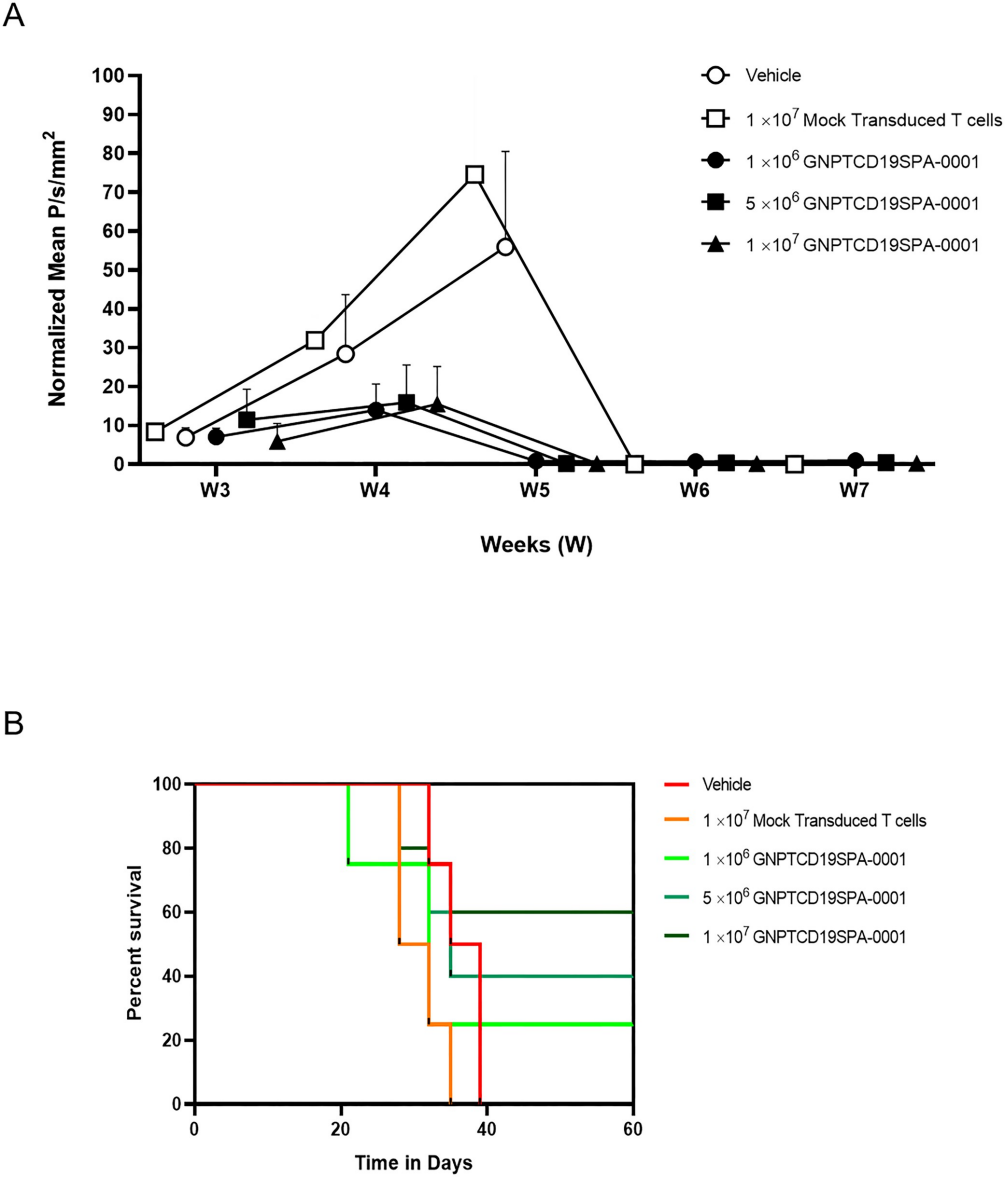
Tumor suppression and percent survival of mice engrafted with Raji cells and treated with CAR-CD19 T cells. Tumor burden in immunocompromised mice engrafted with Raji expressing luciferase as assessed by bioluminescence following treatment with the indicated doses of CAR-CD19 T cells. (A) Normalized bioluminescence intensity was shown are the mean ± SEM. (B) Percent survival of C.B.17/Icr-scid/scidJcl mice engrafted with 5 x 10^5^ Raji cells two weeks before administration of the indicated dose of CAR-CD19 T cells. Note: mice in control groups had been culled due to disease progression prior to week 4. N ≥ 4 mice/group.

An immunodeficient mouse model of CD19-positive lymphoma was used to determine the *in vivo* safety and efficacy of LV52 transduced T cells. Mice (C.B.17/Icr-scid/scidJcl) were injected via the tail vein with 5 x 10^5^ Raji cells expressing LUC and green fluorescent protein (GFP). LV52 transduced T cells were intravenously injected via the tail vein two weeks later at 1 x 10^6^, 5 x 10^6^, or 1 x 10^7^ cells/mouse. Control mice received 1 x 10^7^ mock transduced T cells or vehicles. Tumor burden was assessed by bioluminescence following the injection of D‐luciferin potassium salt (Xenolight) using In vivo Imaging System MX FX PRO (Carestream Health Inc) every week for up to 5 weeks after receiving LV52 transduced T cells (Fig 6A). The results revealed a steady increase in tumor burden in mice receiving mock transduced T cells or vehicles alone. However, mice receiving all doses of LV52 transduced T cells demonstrated a dramatic decline in tumor burden by week three after receiving the CAR-CD 19 T cells.

### Increase survival of CAR-CD19 T cells treated mice

Mice receiving CAR-CD19 T cells appeared to show a dose-dependent increase in the length of survival, with 25%, 40%, and 60% of animals receiving 1 x 10^6^, 5 x 10^6^, or 1 x 10^7^ cells, respectively, surviving until the end of the experiment on day 60 (Fig 6B). In contrast, all animals that received vehicle or mock transduced T cells had been euthanized due to progressive disease by day 38, demonstrating that CAR-CD19 T cells can extend the survival of mice with human CD19-positive tumors.

## Discussion

The efficacy of the gene therapy product often depends heavily on the transduction efficiency of the viral vector and the selected MOI. The lentiviral vector is the most common viral vector used to produce CAR-CD19 T cells [18]. Because of their capacity to stably integrate into the host cell genome and infect both dividing and non-dividing cells, these vectors have been extensively used for gene and cellular therapy [19, 20]. Various generations of lentiviral vectors are created, with an added benefit for each increasing generation [21–23]. The current study aims to test the third-generation lentiviral vector with the proprietary CD19 sequence to determine the optimal MOI and the proof-of-concept efficacy in both *in vitro* and *in vivo* tests. Choosing the MOI of the viral vector to be utilized in the study is essential as using too low MOI can result in a low expression level of the gene of interest. On the other hand, using too high MOI will result in increased copies of the transgene getting integrated into the chromosome of the targeted cells leading to a higher risk of insertional mutagenesis [24].

Employing four different MOI (1, 5, 10, and 20), we have demonstrated a dose-dependent increase in the expression level of the transgene and cytotoxic capability to lyse the target cells of the CAR-CD19 T cells without affecting the viability of the cells. In addition, the transduced CAR-CD19 T cells did not produce an off-target effect and thereby conferred specificity, as evident by the lack of activity in the co-cultures between CAR-CD19 T cells and allogeneic T cells. This data was in agreement with previous studies that confirmed the potent cytotoxicity of CAR-CD19 T cells against CD19+ cancer cells [25–27]. Based on the specificity of our constructed CAR, this may be used to create more effective Tandem CARs, which currently strike the interest to confer a therapeutic advantage both in hematologic malignant and solid tumors [28–31]. Though, the variation observed in measuring the vector copy number of CAR T cells can occur since the samples came from different donors.

Given that T cell-induced IFN-γ secretion usually is tightly associated with antitumor efficacy [32], the increased cytotoxicity observed in the CAR-CD19 T cells in response to Raji cells is consistent with the increase in the concentration of IFN-γ produced in the co-cultures. Our findings confer increased specificity on the antitumor ability of produced CAR-CD19 T cells, which concord with previous demonstrations [33–35]. Likewise, using a third-generation lentiviral vector, CAR can functionally target CD19-expressing cancer cells by cytolysis and by the production of cytokines. The efficiency of transduction by the third-generation lentiviral vector is higher than the control, with an MOI of 20 for the second-generation lentiviral vectors. This finding was apparent with the use of lower MOI (MOI of 5) for the third-generation lentiviral vector to achieve a similar level of expression, lysis activity, and secretion of IFN-γ as the MOI of 20 for the second-generation lentiviral vector. The MOI of 20 for the second-generation lentiviral vector was selected based on the previous study that showed killing properties of the transduced T cells and clinical benefit in one relapsed ALL patients under the compassionate ground utilizing the same CD19 transgene [17]. In the current setting, the MOI of 5 appears to be the optimal level for the transduction of T cells using the third-generation lentiviral vector.

The current study also aimed to examine and further verify the efficacy of engineered CAR-CD19 T cells in tumor-burdened immunodeficient mice. However, there is no true biochemical mouse model of Acute Lymphoid Leukemia (ALL). Therefore, a single dose of tumor cell line (Raji LUC/GFP) was injected into immunodeficient mice (C.B.17/Icr-scid/scidJcl) to create a model as close to the human disease as possible. The dose of the Raji cancer cell line (500,000 cells/mouse) was based on previously published data [36]. Administration of both the Raji cell line and CAR-CD19 T cells did not affect the body weight of the treated mice throughout the study period. This finding suggests that the Raji and CAR-CD19 T cells did not induce a toxic effect in mice.

Furthermore, CAR-CD19 T cells treated mice showed a reduction in the tumor burden and increased survival rate compared to mocked cells treated mice at all dose levels, suggesting that the current doses provide a beneficial effect in mice with human CD19-positive tumors. The amounts of CAR-CD19 T cells were chosen based on the clinical doses tested in the clinical trial for treating patients with ALL. This study is consistent with the previous findings that showed CARs increased the potency of the engineered T cells in a pre-clinical setting [36–40]. Nevertheless, further pre-clinical testing is required, including experiments in NOD SCID gamma mouse (NSG) animals, to ensure the dose and the in vivo activity of modified CAR T cells.

In conclusion, this study showed good efficacy of the CAR-CD19 T cells transduced with a third-generation lentiviral vector in the pre-clinical setting. The data support the further development of a third-generation lentiviral vector carrying the CD19 transgene in the clinical trial for treating ALL patients.
